# Supplementary material for: Replacing bar graphs of continuous data with more informative graphics: are we making progress?
Source: Clin Sci (Lond). 2022 Aug 10;136(15):1139–56. doi: 10.1042/CS20220287 (PMC9366861; doi:10.1042/CS20220287)
Supplement: Supplementary Tables S1-S2 [file CS-2022-0287_supp.pdf]

Supplemental tables for:

Replacing bar graphs of continuous data with more informative graphics: Are we making progress?

Nico Riedel, Robert Schulz, Vartan Kazezian, Tracey Weissgerber

Table S1: Fields of Research classifications included in the study

Field	Code
Biological sciences	600
Biochemistry & cell biology	601
Evolutionary biology	603
Genetics	604
Microbiology	605
Physiology	606
Medical biochemistry & metabolomics	1101
Cardiorespiratory medicine & Hematology	1102
Complementary & alternative medicine	1104
Dentistry	1105
Human movement & sport science	1106
Immunology	1107
Medical microbiology	1108
Neurosciences	1109
Nursing	1110
Nutrition & dietetics	1111
Oncology & carcinogenesis	1112
Ophthalmology & optometry	1113
Pediatrics & reproductive medicine	1114
Pharmacology & pharmaceutical sciences	1115
Medical physiology	1116
Multidisciplinary journals (≥4 categories)	N/A
Uncategorized journals	N/A

Abbreviations: N/A, not applicable.

Table S2: Papers screened by field and year

Field	2010	2011	2012	2013	2014	2015	2016	2017	2018	2019	2020
Biochemistry & Cell Biology	993	987	989	966	996	996	994	998	1000	994	1000
Biological Sciences	999	994	999	992	995	999	996	998	996	992	996
Cardiorespiratory Med. & Haematology	891	789	887	875	968	924	997	994	992	989	998
Complementary & Alternative Med.	305	499	890	991	989	1000	1000	1000	1000	1000	1000
Dentistry	279	347	642	596	709	789	830	829	851	867	912
Evolutionary Biology	544	898	944	944	931	947	956	982	980	1000	1000
Genetics	1000	1000	1000	1000	1000	1000	1000	992	1000	1000	1000
Human Movement & Sports Science	403	456	542	749	824	860	990	946	1000	997	1000
Immunology	873	921	909	997	967	999	999	976	984	999	1000
Medical Biochemistry & Metabolomics	331	437	441	599	902	733	714	784	870	999	1000
Medical Microbiology	877	907	904	916	935	934	993	972	1000	999	1000
Medical Physiology	347	393	647	843	975	996	1000	1000	1000	1000	1000
Microbiology	1000	1000	1000	970	967	1000	1000	1000	1000	998	1000
Multidisciplinary Journals	1000	1000	1000	1000	1000	1000	1000	1000	1000	999	1000
Neurosciences	959	940	933	959	993	994	998	997	998	999	997
Nursing	768	857	941	999	1000	997	997	996	999	993	1000
Nutrition & Dietetics	388	561	711	925	941	964	1000	1000	1000	1000	1000
Oncology & Carcinogenesis	996	992	998	998	999	997	1000	1000	1000	998	999
Ophthalmology & Optometry	608	698	685	689	815	807	932	899	886	931	988
Paediatrics & Reproductive Med.	652	667	818	826	892	914	978	980	987	981	989
Pharmacology & Pharmaceutical Sci.	739	823	836	918	977	959	971	982	997	974	980
Physiology	226	297	434	556	712	799	945	996	999	999	1000
Unclassified Journals	851	855	919	958	954	937	943	908	871	893	998

Scale
200s
300s
400s
500s
600s
700s
800s
900s

Values represent the number of papers identified and screened in each field for each year. Light green shades correspond to lower numbers (typically due to a limited number of publications being accessible in PubMed Central for the specified field and year), whereas dark blue shades correspond to higher numbers. Abbreviations: Med., medicine; Sci., science.
